# Role of 99mTc sulphur colloid lymphoscintigraphy in a rare case of chylothorax and lymphocele formation post esophageal duplication cyst excision

**DOI:** 10.1186/s41824-021-00111-4

**Published:** 2021-09-21

**Authors:** Naveen Yadav, Sameer Taywade, Rajesh Kumar, Arun Prashanth, Rahul Saxena

**Affiliations:** 1 Department of Nuclear Medicine, AII India Institute of Medical Sciences, Jodhpur, Jodhpur, Rajasthan 342005 India; 2 Department of Pediatric Surgery, AII India Institute of Medical Sciences, Jodhpur, Jodhpur, Rajasthan 342005 India

**Keywords:** Chylothorax, Lymphocele, Filtered Tc-99m Sulphur colloid, Lymphoscintigraphy, SPECT-CT

## Abstract

We report a rare case of chylothorax with lymphocele formation post esophageal duplication cyst (EDC) excision in a 2 year old male child. Patient developed chylothorax after excision of EDC. Pleural fluid cytology showed increased triglycerides and cholesterol levels. Filtered Tc-99m Sulphur colloid lymphoscintigraphy showed abnormal radiotracer uptake in the lower thoracic region on right side corresponding to lymphocele on SPECT-CT images with possible site of leak medially. In addition, Tc-99m pertechnetate scan was done to rule out possibility of residual duplication cyst revealed no abnormality. Patient underwent open and en-masse ligation of the duct. Patient recovered completely post-surgery. This case highlights the importance of lymphoscintigraphy with SPECT-CT in the evaluation of patients with post-operative complications of chylothorax with detection of site of chyle leak.

## Introduction

Incidence of esophageal duplication cysts (EDCs) is around 1 in 8200 and is more common in males than females (Zhangn et al. 2013). EDCs are found commonly in thoracic part of esophagus, nearly 30% in posterior mediastinal region and less frequently in cervical and abdominal part. Diagnosis of EDC is usually established before the age of 2 years when the patient develops symptoms (Watanobe et al. 2015). Thoracic duct injury and development of chylothorax after excision of the esophageal duplication cyst is rare but known complication (Benedict et al. 2016; Kapoor et al. 2014). Here we describe a case of 2 year old child in which filtered Tc99m sulphur colloid lymphoscintigraphy and SPECT-CT helped in the diagnosing and localization of the chylothorax site.

## Case report

**Fig. 1:**
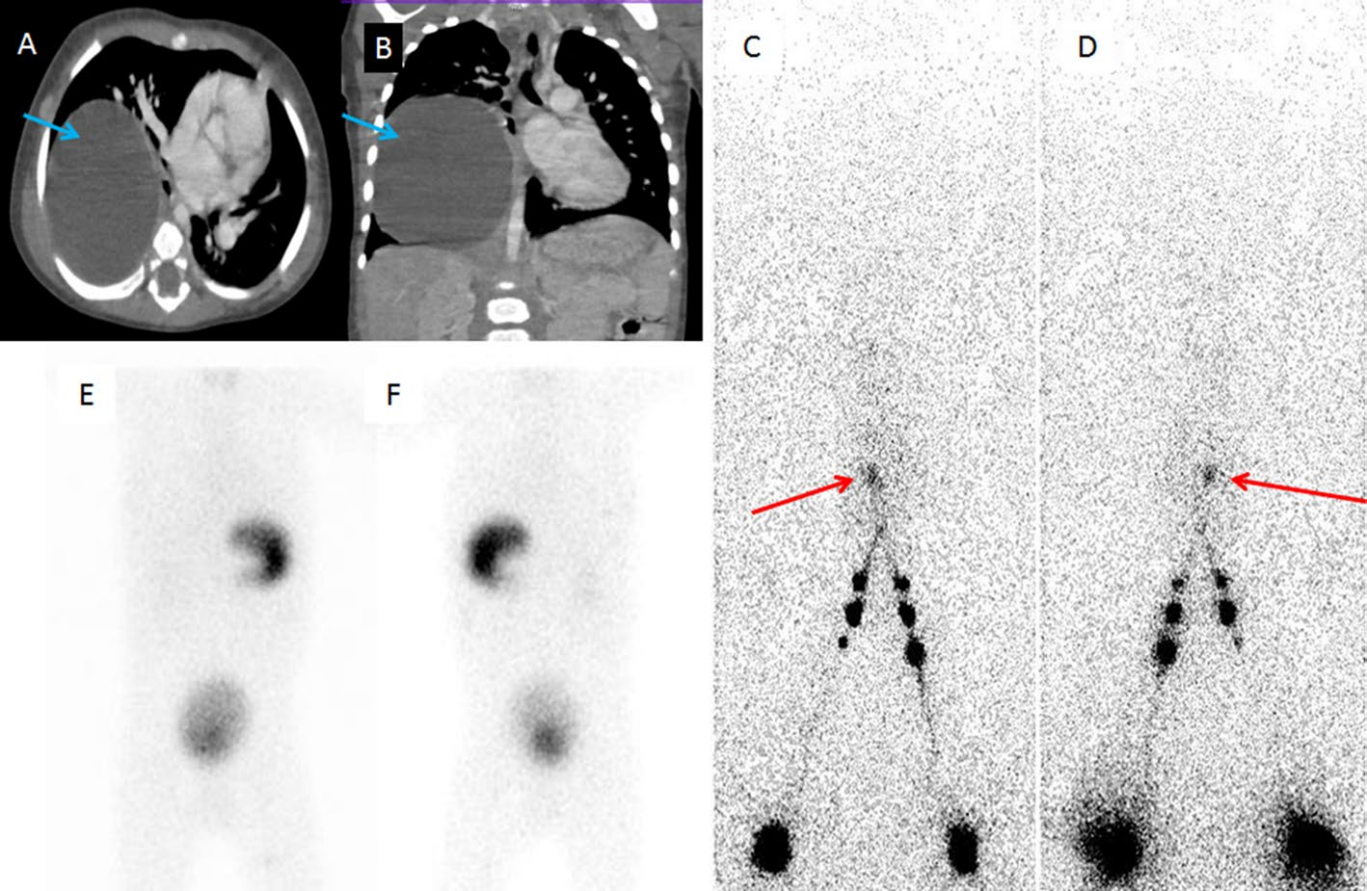
Transaxial and coronal preoperative CT image showing the oval to round shaped large cystic lesion (A,B). Anterior (C) and posterior (D) whole body images acquired 30 min post intradermal injection of filtered Tc99m sulphur colloid in the the first interdigital webs of bilateral lower limbs. Focal accumulation of radiotracer seen in the right lower hemithorax region (as shown by red arrow). No abnormal foci of radiotracer uptake seen in the neck and thorax region on anterior (E) and posterior (F) images acquired after ointravenous injection of 2 mCi of Tc99m pertechnetate to suggest heterotrophic gastric mucosa. Tc99m pertechnetate images were acquired after 48 h of Tc99m sulphur colloid lymphoscintigraphy

A 2-year-old male child presented with fever and pain abdomen for few months. He underwent routine biochemical and hematological investigations for the same. It revealed dyselectrolemia along with hypoalbuminemia, coagulopathy and anemia. His blood parameters were Hb-7.12 gm%, CRP-69.1, TLC-16010, SGOT-1140.8, total protein-4.57 and s.albumin-2.1. His coagulation parameters like PT and INR were 27.71s and 2.2 respectively. Patient further underwent CT thorax and abdomen which showed a large well-defined round to oval shape non- enhancing lesion of size 7.9 × 7.2 × 2.7 cm in right lower hemithorax with split pleura sign and empyema formation (Fig. 1A, B). Considering the age, clinical presentation and biochemical parameters and radiological features, it was diagnosed as an esophageal duplication cyst. In view of anemia blood transfusion was done before the surgery. Subsequently, patient underwent right posterolateral thoracotomy and excision of the duplication cyst. Histopathology of mediastinal mass confirmed foregut duplication cyst. Post-surgery patient developed bradycardia and there was drop in oxygen saturation. Patient was put on ventilator. Intercostal drainage (ICD) tube was placed on right side. ICD tube output showed chylous fluid with triglycerides-91 mg/dl, cholesterol-20.0 mg/dl and WBC-2260/mm^3^. Patient was initially managed conservatively and kept nil per oral for four weeks with total parenteral nutrition (TPN) along with injection octreotide. ICD output decreased and patient improved clinically. But once TPN was tapered off and patient was put on oral diet, output from ICD increased. Clinical condition of patient deteriorated again. There was no improvement in patient condition on conservative management.

**Fig. 2:**
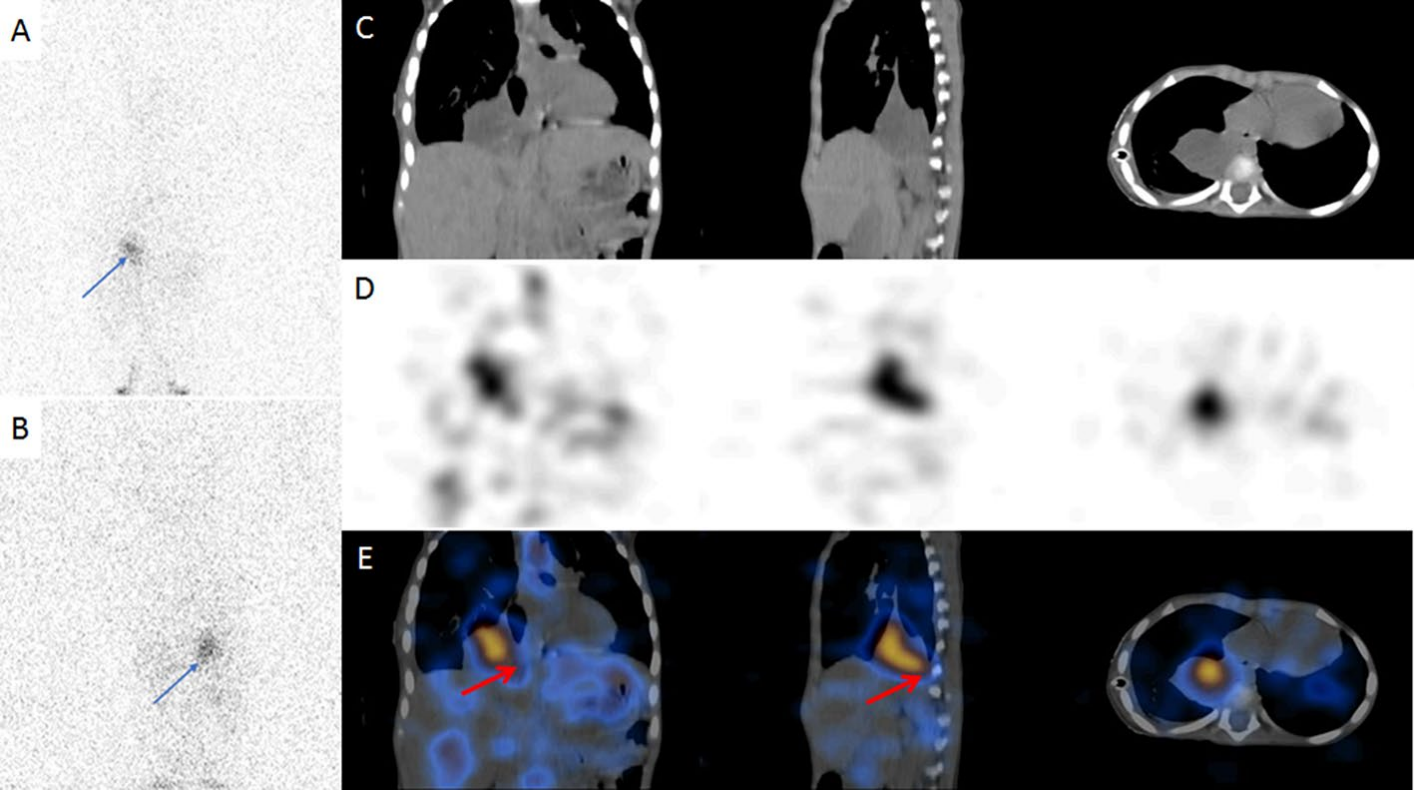
Tc99m sulphur colloid lymphoscintigraphy anterior (A) and posterior (B) delayed planar images of thorax and abdomen region shows focal accumulation of radiotracer in the right hemithorax region (as shown by blue arrow). SPECT-CT images of thorax and abdomen region showing CT (C), SPECT row (D) and hybrid SPECT-CT (E) images in coronal, sagittal and axial thoracic views. Focal radiotracer accumulation corresponding to well-defined cystic area in the right lower thorax at the level of T8-T11 vertebrae with possible site of leak medially (as shown by the red arrow in the coronal and sagittal views) on hybrid SPECT-CT images (E)

**Fig. 3:**
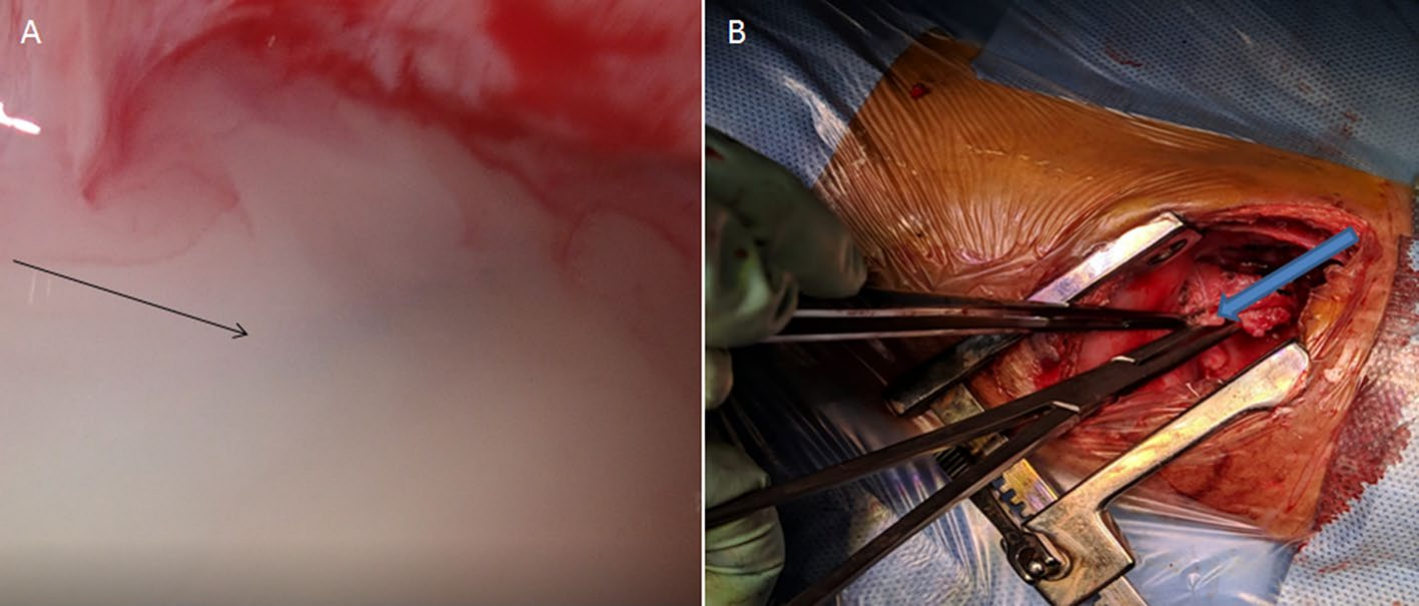
Thoracoscopic/open en masse ligation of thoracic duct surgery performed after [99mTc]sulphur colloid lymphoscintigraphy.(A)Intraoperative images during thoracoscopic surgery showed frank chylous fluid (as shown by black arrow). There was difficulty in identification of thoracic duct from esophagus and aorta during thoracoscopic surgery so procedure converted to open and en masse ligation. B Thoracic duct held by the artery forceps (as shown by blue block arrow). After surgery medium chain triglycerides were started on post-operative day 5

Further, child underwent CT thorax which indicated cystic area in right lower thorax but its nature could not be determined. In view of suspected chylous leak, patient underwent filtered Tc-99m sulphur colloid lymphoscintigraphy. 380 microCi of filtered Tc99m sulphur colloid was injected in two equally divided doses of 190 microCi each in first interdigital web space of bilateral lower limbs intradermally.  Anterior and posterior dynamic whole body planar images were acquired immediately post filtered Tc99m sulphur colloid injections. Static anterior and posterior planar images were acquired at 30 minutes. It revealed focal radiotracer uptake in the region of lower thorax on right side (Fig. 1C, D). SPECT-CT thorax images with low dose CT was also acquired. It revealed focal radiotracer accumulation corresponding to well-defined cystic area in the right lower thorax at the level of T8-T11 thoracic vertebrae with possible site of leak medially (Fig. 2). To eliminate the possibility of residual foregut duplication cyst it was decided to perform Tc99m pertechnetate scan two days after lymphoscintigraphy. 2mCi of Tc99m pertechnetate was injected to the patient and after 20 min neck and thorax imaging was done. Tc99m pertechnetate scan did not demonstrate abnormal radiotracer uptake in the region of cystic lesion in thorax to suggest heterotrophic gastric mucosa (Fig. 1D, E). Based on these two functional imaging modalities, a diagnosis of lymphocele with possible leak site medially was made. Patient was taken for surgery. Initially thoracoscopic surgery was planned but there was difficulty in identification of thoracic duct from esophagus and aorta so procedure was converted to open en masse ligation of the thoracic duct. Intraoperatively, frank chyle was seen from the cystic collection in thoracic cavity confirming a diagnosis of lymphocele (Fig. 3). Post-operatively, started on medium chain triglyceride based diet on post op day-5 (POD) with gradual tapering of TPN. Fluid output from ICD decreased significantly and subsequently ICD tube and Ryle’s tube were removed. Gradually, patient was put on oral diet and discharged from the hospital. On follow up, patient did not develop any symptoms and was doing well.

## Discussion

Diagnosis of EDC is usually established before the age of 2 years when the patient develops symptoms.2 On computed tomography it may present as homogenous lesion with regular margins (Jang et al. 2002). Surgical excision is the mainstay of treatment of EDC. Chylothorax could be one of the post-operative complications associated with EDC.

Causative factors described for development chylothorax can be classified into traumatic and non-traumatic. It could be due to surgical or non-surgical trauma. Whereas, non-traumatic factors include infectious disorder, congenital anomalies, malignancy, miscellaneous disorders or idiopathic. Thoracic duct injury and development of chylothorax after excision of the esophageal duplication cyst (EDC) is rare but known complication (Benedict et al. 2016; Kapoor et al. 2014).

The thoracic duct originates usually at the level of L1 vertebra as a continuation of cisterna chyli. It enters the thorax along the right side of the aorta through the aortic hiatus, in its course at the level of T5 thoracic vertebra it crosses the posterior surface of the aorta to the left, further it continues anterior to scalene muscle in neck, arches to the left and ultimately terminates in the left jugulo-subclavian junction. Thoracic duct injury above the level of T5-6 thoracic vertebra leads to left-sided pleural effusion, while injury below the T5-6 thoracic vertebral level results in right-sided pleural effusion (Merrigan et al. 1997; Bolger et al. 1991; Kettner et al. 1998).

Pleural fluid examination for chylomicrons and triglycerides plays an important role in diagnosing chylothorax. A chylous fluid in pleural effusion typically shows triglycerides levels > l1.1 nmol/l, cholesterol levels less < 200 mg/dl and the presence of chylomicrons (Staats et al. 1980; Seriff et al. 1977; Press et al. 1982). Chylothorax can cause dyselectrolemia, poor nutrition and immunological imbalance in the patient. If leak from thoracic duct is not diagnosed and managed timely it can cause significant morbidity and may even leads to mortality of the patient.

Diagnostic imaging modalities like lymphangiography and lymphoscintigraphy are helpful in confirming chylothorax and detecting the probable site of leak. Lymphangiography, considered as gold standard for chylothorax detection, is an invasive, technically more demanding and has more adverse effects. On the other hand, lymphoscintigraphy is a non-invasive, easy to perform and safe method in evaluating the thoracic duct injury (Pui and Yueh 1998; Sachs et al. 1991). In our case, we used filtered Tc99m sulphur colloid radiopharmaceutical for lymphoscintigraphy. Other agents which have been used for thoracic duct injury detection are serum albumin, Tc99m dextran, aurum-198 (Gold-198), iodine-131 triolein, nanocolloid, oral iodine-123 long-chain fatty acid derivative iodophenyl pentadecanoic acid, or oral iodine-123 heptadecanoic acid (Kettner et al. 1998; Bybel et al. 2001; Sado et al. 2001; Hvid-Jacobsen et al. 1987, 1989; Woolfenden and Struse 1977; Gates et al. 1972; Browse et al. 1997).

In present case, patient developed post-operative complication of chylothorax with continuous leak through ICD. Lymphoscintigraphy with filtered Tc99m sulphur colloid helped in confirming the diagnosis of chyle leak and identifying the possible site of chyle leak as well. In addition, formation of lymphocele was also revealed on SPECT-CT images. In view of above lymphoscintigraphy findings patient’s treatment plan was changed from conservative approach to surgical interventions. Thus, filtered Tc99m Sulphur colloid lymphoscintigraphy proved crucial in the patient management. It not only resolved the clinical dilemma but also gave additional information which could alter the surgical intervention and overall treatment plan.

## Conclusion

Limited data is available in the literature on Tc99m sulphur colloid lymphoscintigraphy SPECT-CT for detection of chylothorax in post esophageal duplication cyst excision. Lymphoscintigraphy is a simple, noninvasive, safe procedure with relatively lesser radiation exposure. It is crucial in confirming the diagnosis of chylothorax, detecting the possible site of leak and identifying other ancillary findings on SPECT-CT which could modify further management. Thus, this case reiterates the importance of lymphoscintigraphy in management of patient with chylothorax.
